# Effect of Bladder Neck Preservation on Long-Term Urinary Continence after Robot-Assisted Laparoscopic Prostatectomy: A Systematic Review and Meta-Analysis

**DOI:** 10.3390/jcm8122068

**Published:** 2019-11-24

**Authors:** Jong Won Kim, Do Kyung Kim, Hyun Kyu Ahn, Hae Do Jung, Joo Yong Lee, Kang Su Cho

**Affiliations:** 1 Department of Urology, Gangnam Severance Hospital, Yonsei University College of Medicine, Seoul 06273, Korea; doctor2play@yuhs.ac (J.W.K.); wharang11co@yuhs.ac (H.K.A.); 2 Department of Urology, Soonchunhyang University Seoul Hospital, Soonchunhyang University Medical College, Seoul 04401, Korea; dokyung80@hotmail.com; 3 Department of Urology, Wonkwang University Sanbon Hospital, Wonkwang University College of Medicine, Gunpo 15865, Korea; haedojung@gmail.com; 4 Department of Urology, Severance Hospital, Yonsei University College of Medicine, Seoul 03722, Korea; joouro@yuhs.ac

**Keywords:** Prostate cancer, radical prostatectomy, bladder neck preservation, urinary continence

## Abstract

This study aimed to evaluate the effect of bladder neck preservation (BNP) on long-term urinary continence after robot-assisted laparoscopic prostatectomy (RALP). We systematically searched the PubMed, Embase, and Cochrane Library databases to identify studies that assessed the difference in urinary continence and oncologic outcomes between patients who underwent RALP with BNP and those who underwent RALP without BNP. Four trials (1880 cases with BNP, 727 controls without BNP) were considered suitable for meta-analysis. BNP was associated with significantly better urinary continence outcomes at 3–4 months (odds ratio (OR), 2.88; 95% confidence interval (CI), 1.52–5.48; *p* = 0.001), 12 months (OR, 2.03; 95% CI, 1.10–3.74; *p* = 0.02), and 24 months (OR, 3.23; 95% CI, 1.13–9.20; *p* = 0.03) after RALP. There was no difference in the rate of overall positive surgical margin (PSM) (OR, 1.00; 95% CI, 0.72–1.39; *p* = 0.99) and that of PSM at the prostate base (OR, 0.49; 95% CI, 0.21–1.13; *p* = 0.09) between the two groups. The BNP technique during RALP leads to early return of urinary continence and long-term urinary continence without compromising the oncologic outcomes.

## 1. Introduction

Radical prostatectomy (RP) is the standard surgical treatment for localized prostate cancer (PCa), with excellent oncologic outcomes [1]. However, surgical complications such as post-prostatectomy incontinence (PPI) and erectile dysfunction are important measures of postoperative outcomes [2] and cause a significant decrease in the quality of life (QoL) of patients [3,4]. Although the recovery of urinary continence depends on various factors, the surgical technique seems to be the most important because it can be improved through the ingenuity and effort of the surgeon. Subsequently, the RP procedure has been continuously refined and various techniques have been introduced and adopted to minimize PPI and improve urinary continence recovery [5,6,7,8,9,10,11].

**Figure 1:**
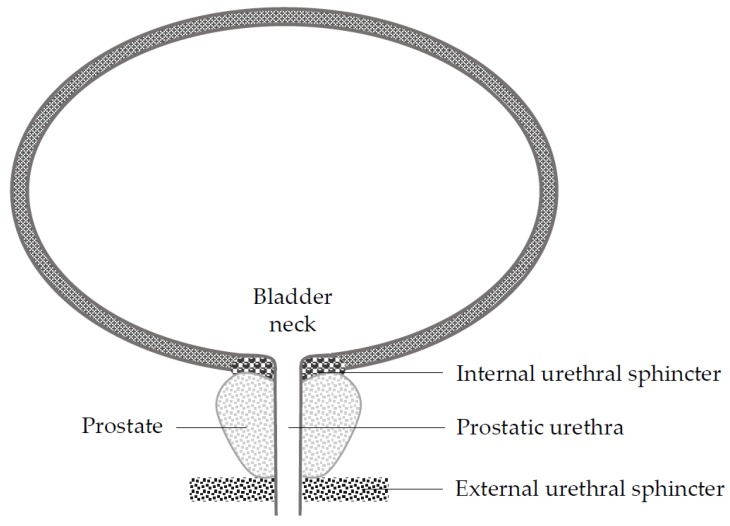
Anatomy of internal and external urethral sphincters. Robot-assisted laparoscopic prostatectomy (RALP) is currently becoming the dominant surgical approach in most countries [24], and RALP was associated with better functional outcomes in some studies [25,26,27]. In this regard, we performed a systematic review and meta-analysis to reappraise the effect of bladder neck preservation (BNP) on early and long-term urinary continence and oncologic outcomes after RALP.

Two functionally independent regions have been identified to be associated with the mechanisms of continence: The distal or external urethral sphincter, and the proximal or internal urethral sphincter located in the bladder neck (Figure 1) [12]. The bladder neck preservation (BNP) technique was developed in an attempt to spare the internal sphincter [13]. Several authors have reported that this technique facilitated urinary continence recovery without compromising cancer control after RP [12,14,15,16,17,18]. Nyarangi-dix et al. presented results of a randomized clinical trial for the effects of bladder neck preservation during radical prostatectomy in 2013 and 2018 [17,18]. In the long term as well as the short term, the continence rate was higher in the BNP group and there was no difference between the two groups in oncologic outcomes such as positive surgical margin (PSM) and recurrence. In addition, they reported that BNP was the only independent predictor of the continence outcome after RP [18]. However, other researchers have suggested that BNP results in little difference in the return of continence but poses a high risk of PSM [19,20,21,22]. Ma et al. [23] recently published a systematic review and meta-analysis showing that patients with BNP during RP had better early and long-term urinary continence than those without BNP. There was no difference in biochemical failure rates between the two groups. However, the authors included all studies regardless of the surgical approach (open, laparoscopic, and robot-assisted).

## 2. Materials and Methods

This systematic review was registered in PROSPERO (CRD42019133381).

### 2.1. Aims of the Study

We aimed to evaluate the effect of BNP on urinary continence and PSM status after RALP.

### 2.2. Search Strategy

We conducted computerized bibliographic searches of the PubMed/MEDLINE, Embase, and Cochrane Library databases up to May 2019. The search terms included “prostate cancer,” “prostatectomy,” “bladder neck,” and “urinary continence or urinary incontinence.” Conference and meeting abstracts were excluded even if they otherwise met the eligibility criteria. The searches identified 769 candidate articles. Two authors (J.W.K. and H.K.A.) independently reviewed the titles and abstracts based on the inclusion criteria, and subsequently reviewed the identified articles.

### 2.3. Study Inclusion Criteria

Following the Preferred Reporting Items for Systematic Reviews and Meta-analyses (PRISMA) guidelines, the eligibility of each study was evaluated using the PICOS (participants, interventions, comparators, outcomes, and study design) method [28]. The study population was defined as adults who underwent RALP with BNP (BNP group) or RALP without BNP (control group). RALP with BNP corresponded to the intervention, whereas RALP without BNP was the comparator. The main outcome was urinary incontinence measured by the count of pad per day (PPD). Urinary continence was defined as the use of “no pad” (0 PPD). The rate of urinary continence was evaluated at various time points (3–4, 12, and 24 months postoperatively). The secondary outcome was the PSM status, and data on overall PSM and PSM at the prostate base were collected. The inclusion criteria with respect to study design were randomized controlled trials and observational studies including cohort and case-control studies. The exclusion criteria were as follows: (1) Editorials and reviews, (2) conference and meeting abstracts, (3) not written in English, (4) not a comparative study, and (5) describing other reconstruction procedures combined with the BNP technique. Studies that met the exclusion criteria were excluded even if they otherwise met the inclusion criteria.

### 2.4. Data Extraction

Two authors (J.W.K. and H.K.A.) reviewed the full articles and extracted the data from each study, independently. Any disagreement with respect to study selection or analysis was resolved through discussion and consultation with a third reviewer (K.S.C.) to reach a consensus. The extracted data included the first author, year of publication, country, study interval, study design, number of patients in the BNP and control groups, baseline characteristics of the study population, outcomes of interest, and information for the assessment of the risk of bias. For outcomes of interest, the numbers of events, odds ratio (OR), 95% confidence interval (CI), and *p*-values were extracted. If data were presented as percentages, raw numbers were calculated.

### 2.5. Assessment of Study Quality

The quality of the included clinical trials was evaluated according to the Newcastle-Ottawa Scale (NOS) [29]. The three major assessment categories of NOS were selection, comparability, and exposure. A study could be granted up to nine stars, and a final score of six stars or more was considered to indicate high quality.

The Grading of Recommendations, Assessments, Developments, and Evaluation (GRADE) system was used to provide a systematic approach to the evaluation of the quality of evidence and the strength of recommendations [30]. The criteria for consideration were assessment of methodology, precision of results, consistency of results, directness, and risk of publication bias. On the basis of these five criteria, we assessed only direct evidence of pairwise meta-analysis by classifying the quality of evidence as one of four levels (i.e., high, moderate, low, and very low).

### 2.6. Statistical Analysis

The present meta-analysis was conducted according to the recommendations of the Cochrane Collaboration and the Quality of Reporting of Meta-analyses guidelines [31]. ORs with 95% CIs were used to evaluate dichotomous variables (urinary continence and PSM). An OR significantly >1.0 favored the BNP group in terms of continence outcomes, whereas an OR significantly <1.0 favored the BNP group in terms of PSM outcomes. All *p*-values are two-tailed, with *p* < 0.05 representing statistical significance.

The quantity of heterogeneity was evaluated using chi-square test and I^2^ statistics, with significance set at *p* < 0.05. In cases in which higher I^2^ and chi-square statistic values indicated increasing inconsistency between studies and significant inter-study heterogeneity, a random-effect model was adopted. Sensitivity analysis was performed by omitting the included studies sequentially and then evaluating the stability of results. Funnel plots and the Egger test of funnel plot symmetry were used to evaluate publication bias. We planned to use funnel plots to assess small study effects in ≥10 studies, however, funnel plot analysis was not implemented because <10 studies were included in the analysis.

Meta-analysis was performed using Review Manager v.5.3 (Nordic Cochrane Center, Cochrane Collaboration, Copenhagen, Denmark, 2008). All *p*-values were two-sided and, except for the test of discrepancy, *p* < 0.05 was considered to indicate a statistically significant result.

## 3. Results

### 3.1. Search Results

**Figure 2:**
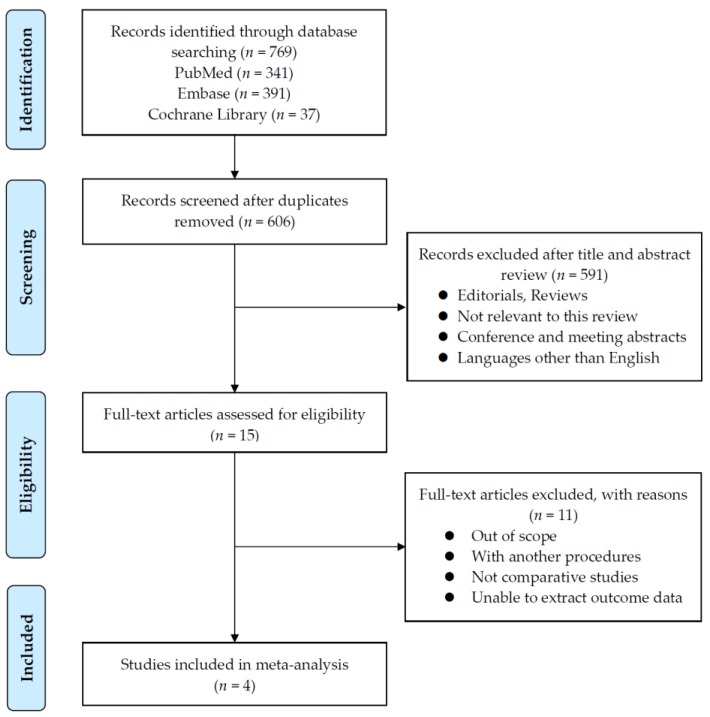
Literature analysis and data acquisition.

A PRISMA flow diagram summarizing the data is shown in Figure 2. We identified a total of 769 studies, of which 606 remained after removing duplicates. Of 606 articles, 591 articles were removed according to the selection criteria. Thereafter, we analyzed the full text of the remaining 15 articles to ensure that they satisfied the inclusion criteria. Four studies were considered suitable for the current meta-analysis, including two prospective and two retrospective observational studies [32,33,34,35]. The data from Freire et al.’s study was a part of Friedlander et al.’s study [32,33], therefore only one of the two studies were adopted for meta-analysis depending on the purpose.

### 3.2. Characteristics of Eligible Studies

The detailed information of each included study is presented in Table 1. A total of 2607 patients were included, comprising 1880 patients with BNP during RALP and 727 patients who underwent RALP without BNP. All of the included studies used a “0 PPD” definition for continence. One of the studies used two definitions (“0 PPD” and “0–1 PPD”) for continence, but only data using the “0 PPD” definition for continence were collected. The timing of continence assessment and reporting ranged from immediately to 1–24 months after surgery.

### 3.3. Urinary Continence at 3–4 Months

**Figure 3:**
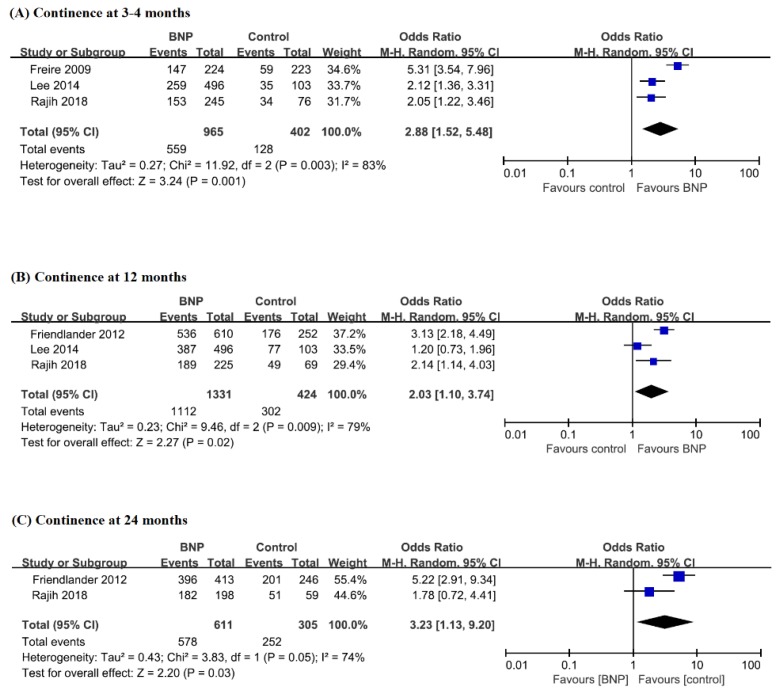
Forest plots for continence at 3–4 months (**A**), 12 months (**B**), and 24 months (**C**) between patients with BNP and those without BNP. BNP, bladder neck preservation; CI, confidence interval.

Three studies demonstrated the continence outcomes at 3–4 months. The urinary continence rate of the BNP group (57.9%) was significantly higher than that of the control group (31.8%) (OR, 2.88; 95% CI, 1.52–5.48; *p* = 0.001) (Figure 3 and Table 2).

### 3.4. Urinary Continence at 12 Months

Three studies showed the continence outcomes at 12 months. The urinary continence rates of the BNP and control groups were 83.5% and 71.2%, respectively (Table 2). The BNP group showed significantly better continence outcomes than the control group (OR, 2.03; 95% CI, 1.10–3.74; *p* = 0.02) (Figure 3).

### 3.5. Urinary Continence at 24 Months

Notably, two studies reported the continence recovery at long-term follow-up (24 months). The continence rate of the BNP group was 94.6% and that of control group was 82.6% (Table 2). There was a statistically significant difference between the two groups (OR, 3.23; 95% CI, 1.13–9.20; *p* = 0.03) (Figure 3).

### 3.6. Oncologic Outcomes

**Figure 4:**
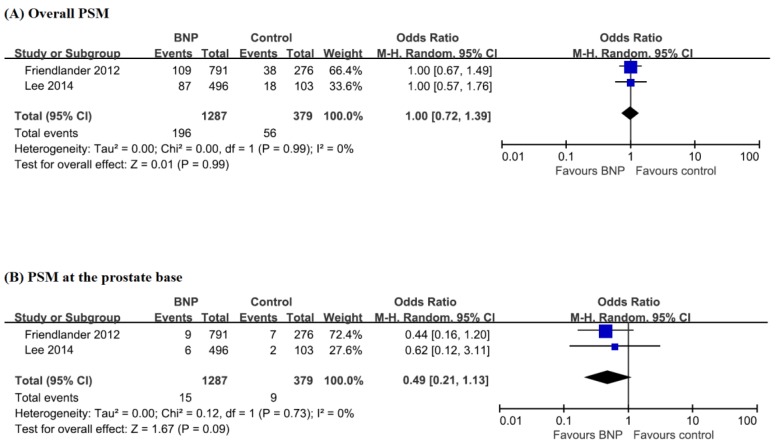
Forest plots for overall PSM (**A**) and PSM at the prostate base (**B**) between patients with BNP and those without BNP. BNP, bladder neck preservation; CI, confidence interval; PSM, positive surgical margin.

There was no significant difference in the overall PSM outcomes (OR, 1.00; 95% CI, 0.72–1.39; *p* = 0.99) and PSM outcomes at the prostate base (OR, 0.49; 95% CI, 0.21–1.13; *p* = 0.09) (Figure 4). Friedlander et al. only reported about biochemical recurrence [33]. There was no difference in biochemical recurrence-free survival between the two groups (hazard ratio, 1.20; 95% CI, 0.62–2.31; *p* = 0.596).

### 3.7. Quality Assessment, Sensitivity, and Publication Bias

The results of the quality assessment of the included studies according to the NOS are shown in Table 3.

Sensitivity analysis was conducted to evaluate the influence of individual studies on the overall meta-analysis results, by omitting one study at a time. Omission of any study made no significant difference, demonstrating that our results are statistically reliable.

The results of the GRADE quality assessment of direct evidence of each comparison are shown in Table 2. The certainty was moderate in three of the comparisons and low in two of the comparisons.

## 4. Discussion

RALP causes both anatomical and functional alterations in the sphincteric mechanism and the relevant supporting structures (pubourethral ligaments, arcus tendineus fascia, endopelvic fascia, Denonvilliers’ fascia, and detrusor slips), and these changes affect urinary continence [36]. In a normal male individual, the sphincteric mechanism is composed of the internal sphincter (bladder neck) proximally, the external sphincter distally, and the connecting longitudinal smooth muscle of the urethra and prostate. The external sphincter is horseshoe shaped, composed of an outer layer of striated muscle and an inner layer of smooth muscle, and believed to be responsible for active urinary continence [36]. The internal sphincter is composed of ring-shaped smooth muscle fibers from the bladder trigone, which surrounds the urethra circumferentially under the control of hypogastric nerves [36,37]. Injury to sphincters and their neural supply can result in PPI. Although there have been various attempts to preserve the continence mechanism, no perfect solution has been found yet. The BNP technique is one of the methods introduced to facilitate urinary continence through the sparing of the internal sphincter by isolating and dissecting the prostatic urethra [13,38]. However, the effect of BNP on urinary continence has been controversial, and there remain concerns about its higher risk of PSM [12,14,15,16,19,20,21,22].

In the current study, we clearly demonstrated that the BNP technique during RALP can improve both short-term and long-term urinary continence outcomes without compromising the oncologic outcomes. A prior meta-analysis by Ma et al. [23] also suggested the beneficial effect of BNP on the same endpoints; however, there are several differences between their meta-analysis and ours. First, they included all studies regardless of the surgical approach (open, laparoscopic, or robotic RP). On the other hand, we analyzed RALP series only, which increases the value of our study in the era of robotic surgery. RALP has been popularized worldwide with the magnified three-dimensional high-definition vision system and miniaturized wristed instruments, which enable microsurgery and the preservation of the most delicate anatomical structures. For these reasons, we attempted to reappraise the role of BNP in improving continence after RALP. The OR for continence rate at 12 months in the current study was 2.03 (95% CI, 1.10–3.74), which seems to be higher than that reported by Ma et al. (OR, 1.46; 95% CI, 1.06–2.02) [23]. These observations suggest that the favorable effect of the BNP technique might be more pronounced when it is performed as a part of RALP.

Second, a strict definition of urinary continence (0 PPD) was applied in the current study, whereas the definition used in Ma et al.’s study [23] was mixed and relatively broad (0, 1, or 2 PPD). The definition of continence is highly arguable and is an important aspect in both research and clinical practice. The most concerning issue is the acceptance of the use of a safety pad. Urinary continence was typically described as 0–1 PPD in the 1990s and thereafter. However, a significant decrease in health-related QoL was observed, even in patients who used only 1 PPD after RP compared with those who used 0 PPD [39], and patients who required 2 PPD had seriously affected QoL [40]. Currently, continence is defined as 0 PPD by most centers, and many reports continue to define continence as 0 PPD without considering the use of a safety pad [41]. In addition, we analyzed the long-term continence results until two years postoperatively, thus representing the longest follow-up data among the meta-analyses assessing the effect of a certain technique on urinary continence. The probability of becoming continent is known to progressively improve after surgery, and the final continence outcome is achieved at about two years postoperatively [40].

There have been several systematic reviews and meta-analyses about other reconstructive techniques for reducing PPI. Posterior musculofascial reconstruction (Rocco’s stitch) is currently one of the most widely used reconstruction methods. Grasso et al. [42] reported a meta-analysis demonstrating that posterior reconstruction during RALP had a significant advantage on urinary continence in the first 30 days (relative risk, 1.60; 95% CI, 1.20–2.12; *p* < 0.0001); however, there was no significant advantage in terms of urinary incontinence after 90 and 180 days. Cui et al. [43] demonstrated that anterior suspension (Patel’s stitch) was also associated with short-term urinary continence, but not with long-term outcomes. Wu et al. [44] performed a meta-analysis to evaluate the efficacy of total reconstruction (anterior plus posterior reconstruction) versus non-total reconstruction of the pelvic floor on the urinary continence rate after RP, with eight robotic and two laparoscopic RP series. There was a significant benefit on urinary incontinence at 52 weeks (OR, 4.10; 95% CI, 1.80–9.38; *p* < 0.001) in addition to short-term outcomes (1, 2, 4, 12, and 24 weeks); however, the continence outcome at longer than 52 weeks was not reported.

Meanwhile, special attention should be paid on oncologic safety parameters such as PSM and biochemical recurrence. Bellangino et al. [45] performed a systematic review and meta-analysis on the surgical margin status after RP with BNP regardless of the surgical approach. They included two randomized clinical trials, seven prospective comparative studies, two retrospective comparative studies, and four case series, published between 1993 and 2015. The overall and base-specific PSM rates ranged between 7% and 36% and between 0% and 16.3%, respectively. The mean base PSM was 4.9% in patients with BNP, but only 1.85% in those without BNP; thus, the authors concluded that BNP during RP might cause an increase in base-positive margins. However, Nyarangi-Dix et al. [18] reported that there was no significant difference in PSM (*p* = 0.77) and biochemical recurrence (*p* = 0.63) between groups with and without BNP. In the current study, BNP had no negative influence on the overall and base-specific PSM in patients who underwent RALP. These observations can be explained by the appropriate patient selection for BNP throughout the detailed review of preoperative information, together with advances in multiparametric magnetic resonance imaging. Meticulous dissection enabled by a robotic system might also be another key reason for such favorable outcomes.

Although results from systematic reviews and meta-analyses can present the best piece of evidence available in the literature, some potential drawbacks must be taken into consideration. The major concern in the current study is related to the nature of non-randomized observational studies, which cannot avoid inherent limitations such as selection bias. Further, continence recovery is not evaluated in all treated cases because some patients were lost to follow-up or the preoperative urinary continence status was not reported. Another critical issue remains the impossibility of controlling for individual surgeon factors, such as surgical techniques and learning curve with varying levels of expertise, despite the same definition of BNP in the studies. In addition, only articles published in English were included and individual patient data were not available for each study, which is the gold standard for meta-analyses. As these limitations might make the results unstable, further studies are needed to investigate the role of the BNP technique in RALP. To better assess the effectiveness of the BNP technique with respect to the early recovery of urinary continence and its long-term superiority, prospective multicenter randomized controlled studies are required. In this study, publications describing the combination of BNP and another procedure were excluded to more clearly elucidate the role of BNP. However, the impact on urinary continence of BNP in conjunction with other techniques, such as anterior, posterior, and total reconstruction, during RALP should also be assessed in the near future.

## 5. Conclusions

The current meta-analysis demonstrated that the BNP technique leads to early return of urinary continence and long-term urinary continence without compromising the oncologic outcomes. These findings should be validated in well-designed randomized clinical trials.

## Figures and Tables

**Table 1:** Characteristics of the selected studies.

Study. 1. Year 2. Country 3. Data Collection	Group	*N*	Age * (Year)	Follow-up * (Month)	PSM Overall ^†^	PSM Base ^†^	Catheter Removal * (Day)	Leakage ^†^	Retention ^†^	BNC ^†^	Continence Outcome 1. Definition 2. Timing of Measurement
Freire, et al. [32] ^††^ 1. 2009 2. USA 3. Prospective	BNP	348	57.1 ± 6.6	12.7 ± 9.7	42 (12.1)	5 (1.4)	7.7 ± 2.44	10 (2.8)	14 (4)	4 (1.1)	1. 0 PPD 2. 4, 12, and 24 months
	Control	271	58.9 ± 6.7	26.7 ± 8.0	37 (13.7)	6 (2.2)	8.0 ± 3.97	4 (1.5)	6 (2.2)	2 (0.7)	
Friedlander, et al. [33] ^††^ 1. 2012 2. USA 3. Prospective	BNP	791	58.9 ± 6.6	25.8	109 (13.8)	9 (1.1)	7.9 ± 3.5	11 (1.4)	NA	NA	1. 0 PPD 2. 12, and 24 months
	Control	276	58.8 ± 6.8	51.7	38 (13.8)	7 (2.5)	8.0 ± 3.5	11 (4.0)	NA	NA	
Lee, et al. [34] 1. 2014 2. USA 3. Retrospective	BNP	496	59.3 ± 6.7	NA	87 (17.5)	6 (1.2)	NA	NA	NA	NA	1. 0 PPD or 0–1 PPD 2. 3 and 12 months
	Control	103	60.0 ± 6.5	NA	18 (17.4)	2 (1.9)	NA	NA	NA	NA	
Rajih, et al. [35] 1. 2018 2. Canada 3. Retrospective	BNP	245	60.6	49 ± 25	NA	NA	NA	NA	NA	NA	1. 0 PPD 2. 1, 3, 6, 12, and 24 months
	Control	77	61.6		NA	NA	NA	NA	NA	NA	

BNC, bladder neck contracture; BNP, bladder neck preservation; NA, not available; PPD, pad per day; PSM, positive surgical margin. *: Data are presented as mean ± standard deviation. ^†^: Data are presented as n (%). ^††^: The data from Freire et al.’s study was a part of Friedlander et al.’s study, therefore only one of the two studies were adopted for meta-analysis depending on the purpose.

**Table 2:** GRADE quality assessment of the evidence of each comparison.

		Certainty Assessment					Number of Patients		Effect		Certainty	Importance
Number of Studies	Study Design	Risk of Bias	Inconsistency	Indirectness	Imprecision	Other Considerations	BNP during RALP	Control	Relative (95% CI)	Absolute (95% CI)		
**Continence at 3–4 months**												
3	observational studies	not serious	not serious	not serious	not serious	strong association	559/965 (57.9%)	128/402 (31.8%)	OR 2.88 (1.52 to 5.48)	255 more per 1000 (from 97 more to 401 more)	Moderate	Critical
**Continence at 12 months**												
3	observational studies	not serious	not serious	not serious	not serious	strong association	1112/1331 (83.5%)	302/424 (71.2%)	OR 2.03 (1.10 to 3.74)	122 more per 1000 (from 19 more to 190 more)	Moderate	Critical
**Continence at 24 months**												
2	observational studies	not serious	not serious	not serious	not serious	strong association	578/611 (94.6%)	252/305 (82.6%)	OR 3.23 (1.13 to 9.20)	113 more per 1000 (from 17 more to 151 more)	Moderate	Critical
**Overall PSM outcomes**												
2	observational studies	not serious	not serious	not serious	not serious	none	196/1287 (15.2%)	56/379 (14.8%)	OR 1.00 (0.72 to 1.39)	0 fewer per 1000 (from 37 fewer to 46 more)	Low	Critical
**PSM outcomes of prostate base**												
2	observational studies	not serious	not serious	not serious	not serious	none	15/1287 (1.2%)	25/379 (6.6%)	OR 0.16 (0.02 to 1.17)	55 fewer per 1000 (from 65 fewer to 10 more)	Low	Critical

GRADE, Grading of Recommendations, Assessments, Developments, and Evaluation; BNP, bladder neck preservation; CI, confidence interval; OR, odds ratio; PSM, positive surgical margin; RALP, robot-assisted laparoscopic radical prostatectomy.

**Table 3:** Results of quality assessment according to the Newcastle-Ottawa Scale.

Author	Selection 1	Selection 2	Selection 3	Selection 4	Comparability A	Comparability B	Exposure 1	Exposure 2	Exposure 3	Scores
Freire, et al. [32]	1	1	1	0	1	1	1	1	0	7
Friedlander, et al. [33]	1	1	1	0	1	0	1	1	0	6
Lee, et al. [34]	1	1	1	0	1	1	1	1	1	8
Rajih, et al. [35]	1	1	1	0	1	0	1	1	0	6
